# Peritumoral Cbl is a strong independent prognostic marker after curative resection of hepatocellular carcinoma

**DOI:** 10.18632/oncotarget.5540

**Published:** 2015-10-13

**Authors:** Ju-Bo Zhang, Bo Zhang, Lei Guo, Zhen-Hai Lin, Xiao-Qiang Li, Kun Guo, Hui-Chuan Sun, Qing-Hai Ye

**Affiliations:** ^1^ Liver Cancer Institute and Zhongshan Hospital, Fudan University, Shanghai, China; ^2^ Key Laboratory of Carcinogenesis and Cancer Invasion, Ministry of Education, Shangai, China

**Keywords:** hepatocellular carcinoma, casitas B-lineage lymphoma, peritumoral microenvironment, epidermal growth factor receptor, prognosis

## Abstract

Growing evidences support the concept that peritumoral microenvironment gene expression is an important element for physicians to make an accurate prognosis. Nonetheless, the correlation between peritumoral ubiquitin ligases and the hepatocellular carcinoma (HCC) survival remains unclear till this present. The expression of intratumoral and peritumoral Casitas B-lineage Lymphoma (cb1) and epidermal growth factor receptor (EGFR) in hepatocellular carcinomas (HCCs) followed by curative resection was assessed by tissue microarray-based immune-histochemistry in two independent cohorts (*n* = 352). Their respective prognostic values and other clinicopathologic factors were then evaluated. The peritumoral cbl density, much higher than that in intratumoral tissue, was an independent prognostic factor for overall survival (*P* < 0.001) and time to recurrence (*P* < 0.001) of HCCs after curative resection. The hazard ratio were 1.587 and 1.689, respectively. However, there was no correlation between intratumoral Cbl and prognosis. The peritumoral Cbl was also associated with prognosis even in HCC subgroups with small tumor size, negative AFP, without microvascular invasion and negative HBeAg. After a thorough analysis pertaining to the key role of Cb1 on ubiquitination and degradation of activated receptor tyrosine kinases, we eventually discovered the negative correlation between peritumoral Cbl and EGFR (*P* = 0.015). Furthermore, the combination of peritumoral Cbl and EGFR serves as a much stronger indicator to make an accurate prognosis, especially during early recurrence (*P* < 0.001). These findings suggest that low expression of peritumoral Cbl and EGFR were positively associated with tumor size, microvascular invasion and patients survival after hepatectomy, highlighting the key role of peritumoral liver milieu in HCC progression.

## INTRODUCTION

Hepatocellular carcinoma (HCC) is ranked fifth among the most common types of cancer in men worldwide. In China, it has earned an infamous reputation of being the “second most fatal killer”. HCC is an extremely deadly threat that has resulted in nearly 383,000 deaths in China, in year 2012; this is more than half of the total number of deaths caused by HCC globally (746,000 deaths) [1]. Since most HCCs are identified when they have progressed to a terminal stage, curative therapies are of limited benefit for these patients [2]. In recent years, hepatic resection is being increasingly implemented to treat HCC due to several factors including: the development of imaging modals, early diagnosis by screening high risk groups with serum alpha-fetoprotein (AFP) combined with ultrasound. Nevertheless, recurrences, especially intrahepatic recurrence, occur in most patients eventually, even after curative resection was conducted [3]. Therefore, it is urgent to explore the possible predisposing factors that might contribute to early recurrences in HCC patients.

The radical shift in the study of tumor biology is attributable to discoveries, which confirm that the tumor microenvironment has a profound influence of tumor microenvironment on tumor initiation and progression, rather than playing a mere supporting role [4, 5]. Oxidation and inflammation, the features of tumor associated microenvironment, can cause damages, which unavoidably trigger hepatocytes mutation in cirrhotic liver tissue [6]. Although the primary HCC is removed completely by curative hepatectomy, the microenvironment that is favorable to HCC initiation and progression still persists [7, 8].

Furthermore, during the liver repair process, quiescent hepatocytes undergo replication with the help of epidermal growth factor (EGF), transforming growth factor α (TGF-α) and hepatic growth factor (HGF) stimulation. The molecular mechanisms, which explain the concepts of: inflammatory and hypoxia response, derailed endocytosis, proliferation, and architectural remodeling of the liver [6], also explain the concepts that are involved in liver regeneration and HCC [9, 10]; this may have important implications pertaining to HCC development and recurrence. Therefore, theoretically speaking, the expression profiles of peritumoral tissue may have equal effective prognostic value to that of tumor profiles for HCCs.

Defective endocytosis of growth factor receptors, characterized by altered ubiquitylation, has emerged as a multifaceted hallmark of malignant cells [11]. Casitas B-lineage lymphoma (Cbl), a member of the family of RING finger ubiquitin ligases (E3), mediates the ubiquitination and degradation of activated receptor tyrosine kinases (RTKs), which in turn puts a halt to the RTK mediated signaling pathway [12]. It is common for tumors to escape from cbl-mediated RKLs degradation. Consequently, RTKs become overly-expressed, and this provides an alternative way to sustain growth-promoting signals.

EGF receptor(EGFR) system is one of the most important substrates of Cbl. It is implicated in multiple tumorigenesis, including HCC [9, 13, 14]. Furthermore, increasing evidences suggest that activation of EGFR in non-malignant cells of the neoplastic microenvironment might also play an important role in cancer progression and the pathogenesis of bone metastases [9]. In the adult liver, EGFR is highly expressed and has been proposed to play an important role during liver development [15], function and regeneration. So far, very little evidence is available to justify that Cbl (present within the tumor microenvironment), is involved in HCC recurrence and metastasis. Besides, Cbl's prognostic value (in particular its prognostic value when combined with EGFR), remains to be elucidated.

The aim of this study is to find out the pattern and frequency of Cbl's expression in HCC and peritumoral liver tissue and its correlation to the EGFR expression. Aside from that, we also aimed to evaluate the hypothesis (having 2 independent cohorts and a total 352 HCCs): “low Cbl density in peritumoral liver tissue maintains the EGFR mediated growth-promoting signals, which in turn facilitates the growth of dormant or residually histologic HCC, that results in poor clinical outcomes”.

## RESULTS

### Cbl density in HCC and peritumoral liver tissue

**Figure 1:**
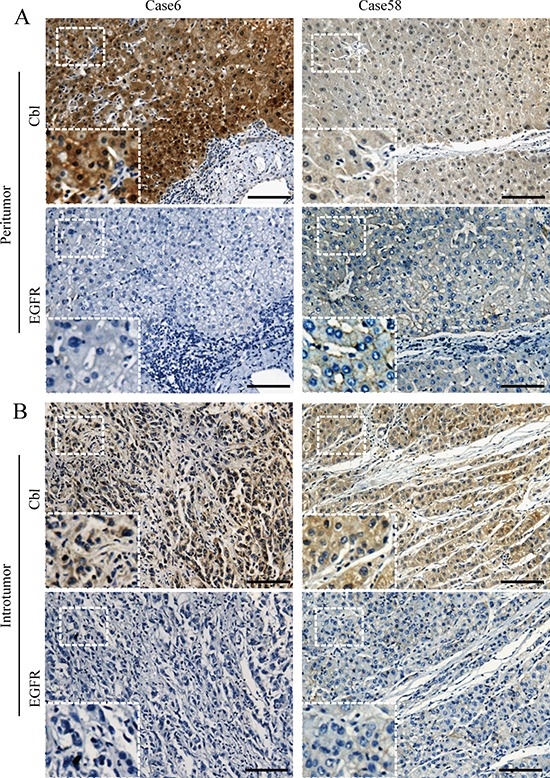
Representative photographs of immunostaining of Cbl and EGFR in tissue microarray **A.** Patients 6 showed high level of Cbl and low level of EGFR in peritumoral liver tissue. On the other hand, patients 58 showed reverse trends. **B.** The expression of intratumoral Cbl and EGFR in these two patients were shown. Scale bars: 100 μm.

**Figure 2:**
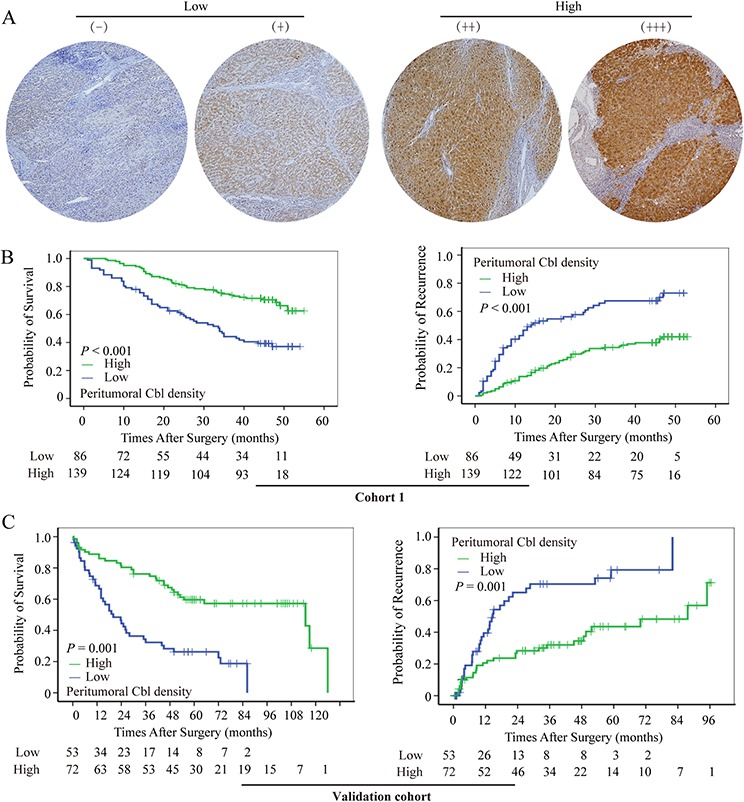
Cumulative overall and time-to-recurrence survival curves of patients with low and high peritumoral Cbl **A.** Tissue microarray cores illustrate the heterogeneity in Cbl density in peritumoral tissue. High peritumoral Cbl level was associated with prolonged OS and TTR in cohort 1 **B.** and validation cohort **C.**

**Figure 3:**
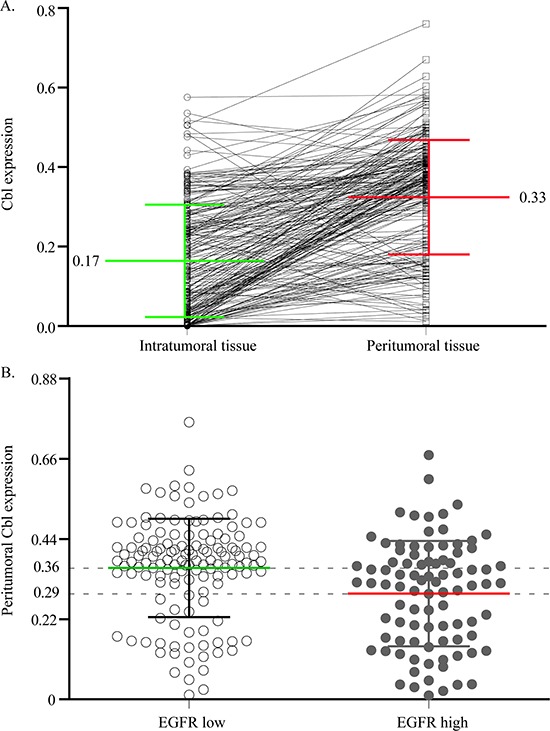
The correlation of Cbl between intratumoral and peritumoral tissue, or peritumoral EGFR low and high expression cases Plot representation of scores according to the cytoplasmic immunohistochemical density of Cbl in intratumoral and peritumoral tissue **A.** and in peritumoral EGFR low and high expression cases **B.** The scores are calculated by the ratio of the IOD/total area.

Cbl protein levels of 227 HCC specimens from cohort 1 were analyzed using IHC. A total of 196 patients (86.3%) were Hepatitis B virus (HBV) surface antigen positive and 82 (36.1%) patients were HBV e antigen (HBe-Ag) positive. During the IHC process, two samples were unexpectedly detached from TMA, leaving insufficient tissue sample behind for the scoring process. Cbl was observed mainly in the cytoplasm of tumor cells and peritumoral hepatocyte, and was usually weak to absent in most stromal cells (Figure 1A, Figure 2A). The comparison between arrayed sections and full sections of Cbl showed that the rate of nonrepresentation for the TMA was less than 5%, giving an efficiency of more than 95% for the 225 peritumor samples (Supplementary Table S1). The average levels of peritumoral Cbl is 0.33, which is much higher than the level in HCC (0.017, *P* < 0.001, Figure 3A, the protein level of Cbl was represented as the ratio of the IOD/total area). All the specimens were stratified into high Cbl level (Cbl_hi, the ratio ≥ 0.33) and low Cbl level (Cbl_lo, the ratio < 0.33), and 139 specimens (61.8%) belonged to the Cbl_hi group for peritumoral Cbl stratification.

**Table 1:** Univariate and multivariate analysis of factors associated with survival and recurrence

Features	Overall survival				Time to recurrence			
	Multivariate				Multivariate			
	Univariate, *P* value	Hazard Ratio	95% CI	*P* value	Univariate, *P* value	Hazard Ratio	95% CI	*P* value
Hepatitis B e antigen: positive vs. negative	0.038	1.543	1.027–2.317	0.041	0.013	1.587	1.077–2.340	0.020
AFP: >400 vs. ≤400 ng/ml	0.024			NS	0.005			NS
Tumor size: >5vs. ≤5 cm	<0.001	3.097	1.982–4.840	<0.001	<0.001	1.842	1.226–2.766	0.003
Microvascular invasion: yes vs. no	<0.001	2.035	1.341–3.088	0.001	0.001	1.487	1.008–2.193	0.045
Intrahepatic metastasis: yes vs. no	0.004			NS	0.008	1.791	1.079–2.972	0.024
Tumor differentiation: low vs. high	0.027			NS	0.235			NA
TNM stage: IIIa vs. II vs. I	<0.001			/	<0.001			/
Intratumoral c-Cbl level: low vs. high	0.608			NA	0.429			NA
Peritumoral c-Cbl level: low vs. high	<0.001	1.587	1.039–2.426	0.033	<0.001	1.689	1.134–2.516	0.005
Intratumoral EGFR level: high vs. low	0.649			NA	0.671			NA
Peritumoral EGFR level: high vs. low	<0.001			NS	<0.001	1.689	1.134–2.516	0.010

Note. Univariate analysis, Cox proportional hazards regression model.

Abbreviations, NA, not adopted; NS, not significant; AFP: alpha fetoprotein; ALT

Cbl status among the 225 HCCs was compared with clinical parameters (Supplementary Table S2). Cbl staining patterns were similar irrespective of liver cirrhosis, tumor encapsulation, differentiation, and intrahepatic metastasis. Patients with large tumor size, high AFP level, microvascular invasion and HBeAg positive were more likely to be peritumoral Cbl_lo (Supplementary Figure S1), whereas, intratumoral Cbl density did not demonstrate any correlation to any clinicopathologic features.

### The prognostic value of peritumoral Cbl density for HCCs in univariate and multivariate analysis

**Figure 4:**
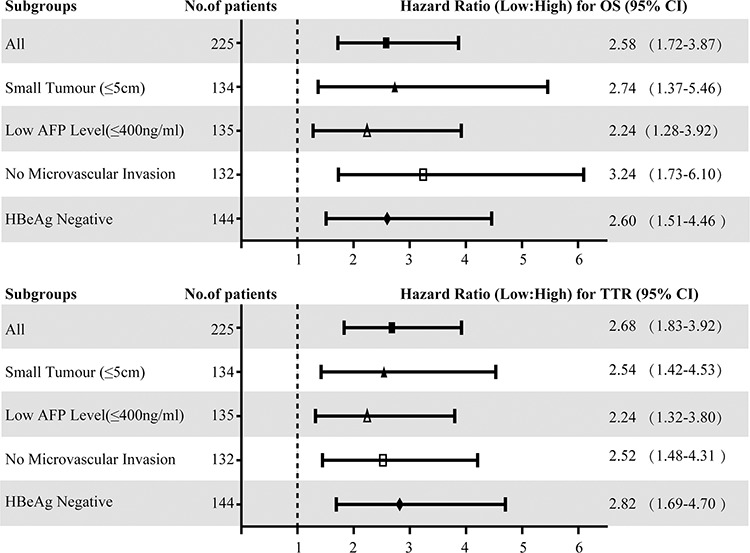
Hazard ratios (HR) of peritumoral Cbl for OS and TTR in different subgroups of HCC patients with curative resection The HR of peritumoral Cbl density for OS and TTR in each subgroup were calculated by comparing the patients with low Cbl density with those with high Cbl density. HR > 1.0 indicates a worse outcome. The median Cbl density in peritumoral liver tissue was used as the cut-off value.

At the time of the last follow-up, 108 patients had tumor recurrence, and 95 patients had died, including 18 patients who died of liver failure and other reasons without record of tumor recurrence. the 1-, 3-, and 5- year overall survival (OS) rates were 89%, 63%, and 51%, respectively; and 1-, 3-, and 5- year recurrence rates were 25%, 47%, and 53%, respectively. By Kaplan-Meier analysis, peritumoral Cbl_lo group had a significantly worse OS compared to the peritumoral Cbl_hi group in cohort 1 (LR = 22.7, *P* < 0.001, Figure 2B, left panel). The median OS was 33 months, and there were 52 deaths in 86 patients of peritumoral Cbl_lo group compared with median survival of >55 months and 43 deaths in 139 patients of peritumoral Cbl_hi group. In addition, peritumoral Cbl was associated with time to recurrence (TTR) and the mean TTR was 14 months in Cbl_lo group but >55 months in Cbl_hi group (LR = 28.1, *P* < 0.001, Figure 2B, right panel). The univariate Cox proportional HR of peritumoral Cbl density was 2.58 for OS, and 2.68 for TTR (Figure 4). However, Cbl density in intratumoral tissue was not significantly associated with OS (*P* = 0.608) or TTR (*P* = 0.429) (Supplementary Figure S2a–2b). These results were further validated in another cohort comprised of 125 postoperative HCC patients (cohort 2) with more than 10-years of follow-up data (Supplementary Table S3). The peritumoral Cbl density of which can also predict HCC patients’ OS (*P* < 0.001) and TTR (*P* < 0.001) (Figure 2C).

Factors showing significance in prognosis of HCCs by univariate analysis were adopted when multivariate Cox proportional hazards analysis were performed. Compared with patients with peritumoral Cbl_hi, the adjusted Cox proportional HR for peritumoral Cbl_lo patients was 1.587 (*P* = 0.033) and 1.689 (*P* = 0.005) for OS and TTR, respectively. In the multivariable model, HBeAg positive, large tumor size, and microvascular invasion all independently and significantly increased both recurrence and mortality of HCC (Table 1).

**Figure 5:**
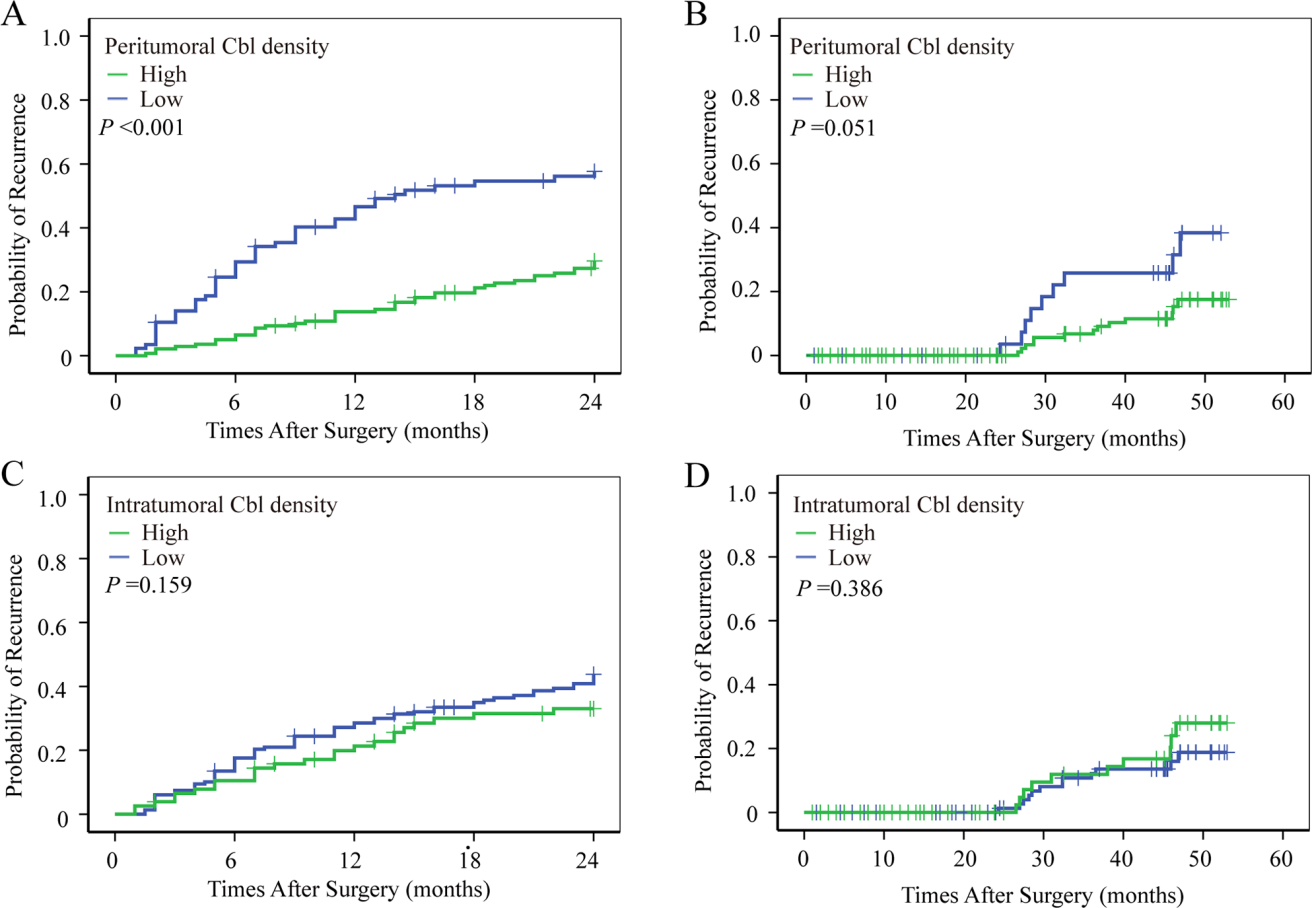
The prognostic value of peritumoral Cbl for early recurrence Using 2 years as a cutoff value, postoperative recurrence was discriminated into early and later recurrence according to the TTR. Patients with low peritumoral Cbl density more tend to have early recurrence **A–B.** While early of late recurrence did not differ between patients with high and low intratumoral Cbl density **C–D.**

As recurrence rate is high in HCC even after curative resection, early prediction and detection of recurrence is clinically important for improving the prognosis. It has been suggested that there are two biologically different recurrence forms (early recurrence and late recurrence) for HCCs, and early recurrence usually occurs within 2 years after treatments. We conducted a further study to demonstrate the value of peritumoral Cbl by predicting early recurrence of HCC and found out that patients with low peritumoral Cbl density tended to have early recurrence (*P* < 0.001), while intratumoral Cbl density was not associated with early recurrence (Figure 5).

### The predictive value of peritumoral Cbl in clinical subgroups of HCC patients

As mentioned above, there was a significant correlation between low peritumoral Cbl and the following factors: large tumor size, microvascular invasion, HBeAg positive and high AFP level; these factors were in turn significantly associated with worse prognosis of HCC patients. We then attempt to further investigate the prognostic value of peritumoral Cbl in HCC subgroups with small tumor (maximum diameter ≤ 5 cm, *n* = 134), low AFP level (≤ 400 ng/ml, *n* = 135), no microvascular invasion (*n* = 132) and negative HBeAg (*n* = 144). The statistical results showed that the level of peritumoral Cbl was significantly associated with OS and TTR in these subgroups (Supplementary Figure S3). The univariate Cox proportional hazards regression of peritumoral Cbl density was 2.74 for OS, and was 2.54 for TTR in small HCC subgroup. The similar results were also found in the low AFP subgroup (for OS: HR = 2.24, for TTR: 2.24), no microvascular invasion subgroup (for OS: HR = 3.24, for TTR: 2.52) and HBeAg negative subgroup(for OS: HR = 2.60, for TTR: 2.82) (Figure 4). The further adjusted COX proportional hazard regression analysis showed that among small HCC subgroup, only the low peritumoral Cbl level predicted poor TTR (HR = 2.42, *P* = 0.003).

### The expression of EGFR in TMA and its association with Cbl density

Increasing evidences show that Cbl negatively regulate the expression of EGFR [16], therefore, immunolabeling for EGFR was performed on the entire cohort 1 in an effort to determine if the prognostic value of Cbl density was related to EGFR status. We found relatively lesser peritumoral EGFR expression in 136 of 225 (60.4%) patients and in 124 of 225 (55.1%) for intratumoral EGFR expression. Statistical results showed that peritumoral Cbl level was negatively correlated with peritumoral EGFR level in the whole cohort 1 (Figure 3B), or in the subgroups of TNM I-III stages (Supplementary Figure S4). The correlation between EGFR and clinicopathologic factors were shown in Supplementary Table S4. The median OS and TTR for patients with high peritumoral EGFR level were 34.5 months and 18 months, respectively, which were statistically shorter than the median OS and TTR for patients with low peritumoral EGFR level (>45 months and >44 months, respectively; *P* < 0.001 both, Supplementary Figure S5). However, intratumoral EGFR level was not associated with OS and TTR (*P* = 0.649 and *P* 0.671, respectively, Supplementary Figure S2).

### Combination of peritumoral Cbl and EGFR expression further increase the prognostic value

**Figure 6:**
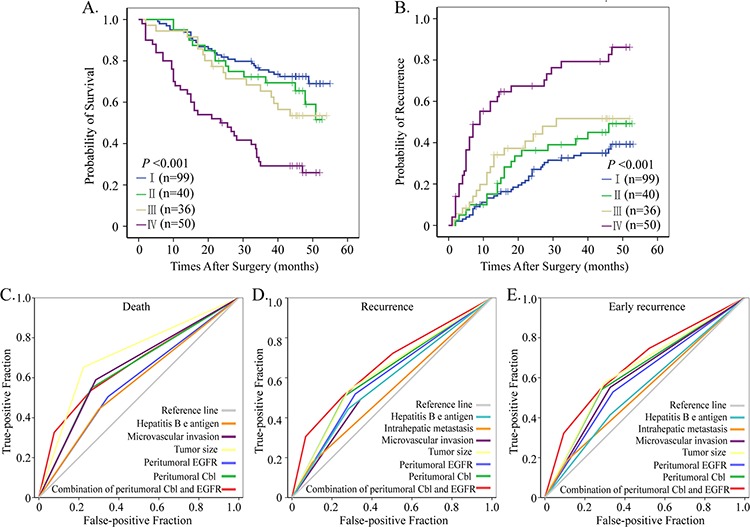
Prognostic and predictive value of combining peritumoral Cbl and EGFR Cumulative overall and recurrence-free survival curves of the combination of peritumoral Cbl and EGFR **A, B.** All factor adopted in receiver operating characteristic analysis predicted death **C.** recurrence **D.** and early recurrence **E.** precisely during follow-up (*P* < 0.05 for all). The predictive value of this combination was the best one for predicting early recurrence.

After a thorough analysis on the negative regulation of Cbl on EGFR and the negative correlation between peritumoral Cbl and EGFR in our study, we decided to classify all the HCC patients in cohort 1 into four subgroups according to their peritumoral Cbl and EGFR levels: subgroup I (*n* = 99, Cbl↑ and EGFR↓); subgroup II (*n* = 40, Cbl ↑ and EGFR ↑); subgroup III (*n* = 36, Cbl ↓and EGFR↓); subgroup IV (*n* = 50, Cbl↓and EGFR ↑). There were significant differences in both OS (*P* < 0.001) and TTR (*P* < 0.001) among the four subgroups (Figure 6A–6B). Notably, 5- year OS and TTR rates in subgroup I were 66% and 61%, respectively, but only 27% and 14%, respectively, in subgroup IV (Supplementary Figure S6).

The combination of peritumoral Cbl and EGFR were adopted and their predictive values were then studied by ROC analysis (Figure 6C–6E). The statistical results showed that peritumoral Cbl alone could predict death and recurrence precisely, the area under the curve (AUC) were 0.643 (*P* < 0.001) for death and 0.622 (*P* = 0.02) for recurrence, respectively. The combination of peritumoral Cbl and EGFR further increased the AUC to 0.678 (*P* < 0.001) for death and 0.658 (*P* < 0.001) for recurrence. Even for prediction of early recurrence, the AUC of combination of peritumoral Cbl and EGFR reached *a* value of 0.660 (*P* < 0.001), which was the most powerful one among all adopted factors. The specific values of all predicted factors were seen in Supplementary Table S5.

## DISCUSSION

Increasing evidences suggest that microenvironment modulation is an important step when it comes to human cancers’ initiation and progression stages, furthermore, it also plays a crucial role in cancer treatment [2, 4, 5, 17]. Recently, both Golub's report [2] and our studies [8, 18] suggest that gene expression signature correlated with survival and recurrence is present in the liver tissue adjacent to the tumor in patients with HCC. In the TMA analysis of 352 HCCs (2 independent cohorts), this study verifies that the expression of peritumoral Cbl has a negative correlation to the expression of peritumoral EGFR. Low levels of Cbl and high expression of EGFR in the peritumoral liver tissue, but not in tumor tissue, was significantly associated with high incidence of recurrence and poor overall survival of HCC after curative resection. Furthermore, the combination of peritumoral Cbl and EGFR makes it very possible to predict patients’ outcomes, especially early recurrence, with greater accuracy and affirmation.

RTKs regulate diverse functions in normal cells, and ubiquitin ligase Cbl, mediating multiubiquitylation of RTKs, is the key molecule to sustain the dynamic balance of RTKs [19, 20]. One of the best-studied examples of how Cbl proteins affect receptor trafficking, and therefore the lifetime of an activated receptor complex, is the regulation of the EGFR by Cbl [21]. It has become a well-established fact that overexpression of EGFR in tumor is common in many types of cancer and is associated with poor outcomes of them [13, 14, 22, 23]. However, activation of the EGFR in non-malignant cell populations of the neoplastic microenvironment might also play an important role in cancer progression. Multiple oncogenic alterations underlie defective endocytosis, and the mutation and downregulation of Cbl were unavoidable under oncogenic pressure [11, 24]. In the present study, we discovered that the expression of Cbl in HCC is down regulated significantly compared to the level of Cbl in peritumoral liver tissue. The peritumoral EGFR level in HCCs with low peritumoral Cbl density was much higher than those with high peritumoral Cbl density.

Much different from the association between intratumoral Cbl and clinical parameters, the expression of peritumoral Cbl was significantly low in HCC patients with large tumor, high AFP level, microvascular invasion and HBeAg positive. This indicates that the cirrhotic liver tissue (soil) with low Cbl density is favoring the growth and metastasis of HCC (seeds). On the other hand, the low expression of Cbl in peritumoral liver tissue may be attributable to the interaction between HCC and the cirrhotic liver tissue. In the present study, peritumoral Cbl and EGFR expression were associated with tumor recurrence and survival, however, the role of intratumoral Cbl and EGFR on HCC recurrence and survivability was weakened after hepatectomy. Many kinds of growth factors were secreted after hepatectomy, in particular, to activate the RTKs signal pathway, which favors the growth of dormant or residually histologic HCC. This is undoubtedly one of the common reasons behind early recurrence. [25, 26] In this microenvironment, low Cbl density decreases the EGFR degradation and increase the VEGF expression, providing a fertilized soil for subclinical metastatic tumor cells. The adjusted COX proportional hazard regression analysis showed that peritumoral Cbl level predicted poor OS and TTR precisely, suggesting the multifaceted roles of Cbl on transforming microenvironment.

The phenomena of “occult cancer” existing in a long term before clinically detected [27] is found in prostate [28], lung [29] and liver [30]. Hepatocytes have a high regeneration capacity due to an abundant network of growth factors and their receptors [31], particularly, EGF/EGFR and TGF-α/EGFR signaling [6, 15]. High expression of hepatocyte-derived monocyte chemotaxis protein-1 (MCP-1), regulated by EGFR mediated Src-Jak2-STAT3 signaling, [32–34], is a potent chemoattractant for macrophage and hepatic stellate cells. As a result, this causes macrophage and hepatic stellate cells to accumulate in peritumoral liver tissue. High density of macrophage in peritumoral liver tissue promotes HCC recurrence and metastasis by: supporting proliferation and survival of malignant cells, promoting angiogenesis, subverting adaptive immune responses, and altering responses to hormones [8, 32]. Following the downregulation of Cbl in peritumoral liver tissue, the EGFR level increased sharply owning to decreased ubiquitylation and degradation of EGFR, which in turn promotes angiogenesis and inflammatory cells infiltration, thus favoring dormant neoplasm progression ultimately [27].

In summary, the present study found low peritumoral Cbl density, associated with high expression of EGFR in peritumoral liver tissue, could predict the postoperative recurrence and survival of HCCs. In addition, the combination of peritumoral Cbl and EGFR further increases the prognostic value, especially for early HCC recurrence. This finding indicates a field effect, since environmental exposure increases the risk or tendency for future malignant transformation and tumor recurrence. More effective strategies should be implemented to enhance anticancer therapies that are capable of normalizing the tumor microenvironment in those HCC patients who are at high risk of recurrence after hepatectomy.

## MATERIALS AND METHODS

Tumor specimens (2 independent cohorts with a total of 352 HCCs) were randomly collected after curative resection from February 2005 to November 2006 and from January 1999 to December 2003 respectively, in the Liver Cancer Institute, Zhongshan Hospital, Shanghai, China. The patient enrollment criteria and postsurgical patient surveillance were similar to those specified in our previous reports [8]. Simply put, patients who were enrolled into this research, must fulfill the following criteria 1) definitive HCC diagnosis and stage by pathology on the basis of International Union Against Cancer Tumor Node Metastasis Classification System(7^th^ edition); 2) no prior anticancer treatment; 3) surgical resection with cut surface being freed from cancer by histological examination; 4) availability of complete clinicopathologic and follow-up data (Supplementary Table S2). Ethical approval for human subjects was obtained from the Research Ethics Committee of Zhongshan Hospital, and each participant has provided written informed consent to participate in this research.

### Follow-up and postoperative treatment

Patients were re-assessed once every 2 months in the first postoperative year, and then re-assessed once every 3–4 months after the postoperative year, at the very least. Follow-up was terminated on March 30, 2010. The longest follow-up was 55 and 126 months in cohort 1 and validation cohort, respectively. Most patients died of intrahepatic recurrence, distal metastasis, or complicated liver cirrhosis. Follow-up procedures were described in our previous study [8]. Treatment modalities after relapse were administered according to a uniform guideline as previously described [8, 35].

### Tissue microarray and immunohistochemistry

The construction of tissue microarray (TMA) and immunohistochemistry (IHC) protocols were detail described in our previous study [8]. Primary antibodies were Cbl (1:200, monoclonal; Cell Signaling Technology, Danvers, MA, USA) and EGFR (1:200, polyclonal; Santa Cruze Biotechnology, Santa Cruz, CA, USA). Before they were used on the arrays, the antibodies were titrated against normal controls and the concentration determined to give optimal sensitivity and specifity in the control tissue. Negative controls were treated identically but with the primary antibodies omitted (Supplementary Figure S7).

### Evaluation of immunohistochemical findings and cutoff values

For Cbl, the density of positive staining was measured using a computerized image system as described previously [8]. Briefly, under high-power magnification (×200), 4 photographs of the representative fields of each dot were captured by a computerized image system composed of a Leica CCD camera DFC420 connected to a Leica DM IRE2 microscope (Leica Microsystem Imaging Solutions Ltd, Cambridge, United Kingdom) with Leica Qwin Plus v3 software, and analyzed with Image-Pro Plus version 6.2 software (Media Cybernatics Inc, Bethesda, MD). A uniform setting was applied to all the slides in order to reduce the errors that might be caused during the data capturing process. Integrated optical density (IOD) of all positive staining of Cbl in each photograph was measured, and its ratio to total area of each photograph was calculated as Cbl density. Those with more than median value were stratified into high expression dots. On the other hand, for EGFR, most the positive staining areas are focused on the plasma membrane. Therefore, it is inappropriate to measure the ratio of positive staining to total area. Instead, the result for the EGFR was determined based on the grading standards of 3 researchers, who were blinded to prior knowledge of clinical and pathological parameters and follow-up data. Those EGFR with definite plasma membrane expression were stratified into high expression dots.

### Statistical analysis

Analysis was performed using the statistical package SPSS for Windows (Version 13.0; SPSS Inc, Chicago, IL). The relationship between immunoreactive markers and clinicopathologic variables was analyzed using the Pearson Chi square test or the Fisher exact test. Quantitative variables were analyzed by the independent-samples *t* test. Overall survival (OS) and time to recurrence (TTR) were assessed using the Keplan-Meier method and then compared with the log-rank test. Univariable risk ratios with 95% confidence intervals(CIs) were calculated using Cox proportional hazards regression(HR) models with forward stepwise selection. Receiver operating characteristic (ROC) curve analysis was used to determine the the predictive value of the parameters. *P* < 0.05 was considered statistically significant.

## SUPPLEMENTARY FIGURES AND TABLES


