# Supplementary material for: Peritumoral Cbl is a strong independent prognostic marker after curative resection of hepatocellular carcinoma
Source: Oncotarget. 2015 Oct 13;6(37):40223–34. doi: 10.18632/oncotarget.5540 (PMC4741890; doi:10.18632/oncotarget.5540)
Supplement: Supplementary file 1 [file oncotarget-06-40223-s001.pdf]

SUPPLEMENTARY FIGURES AND TABLES

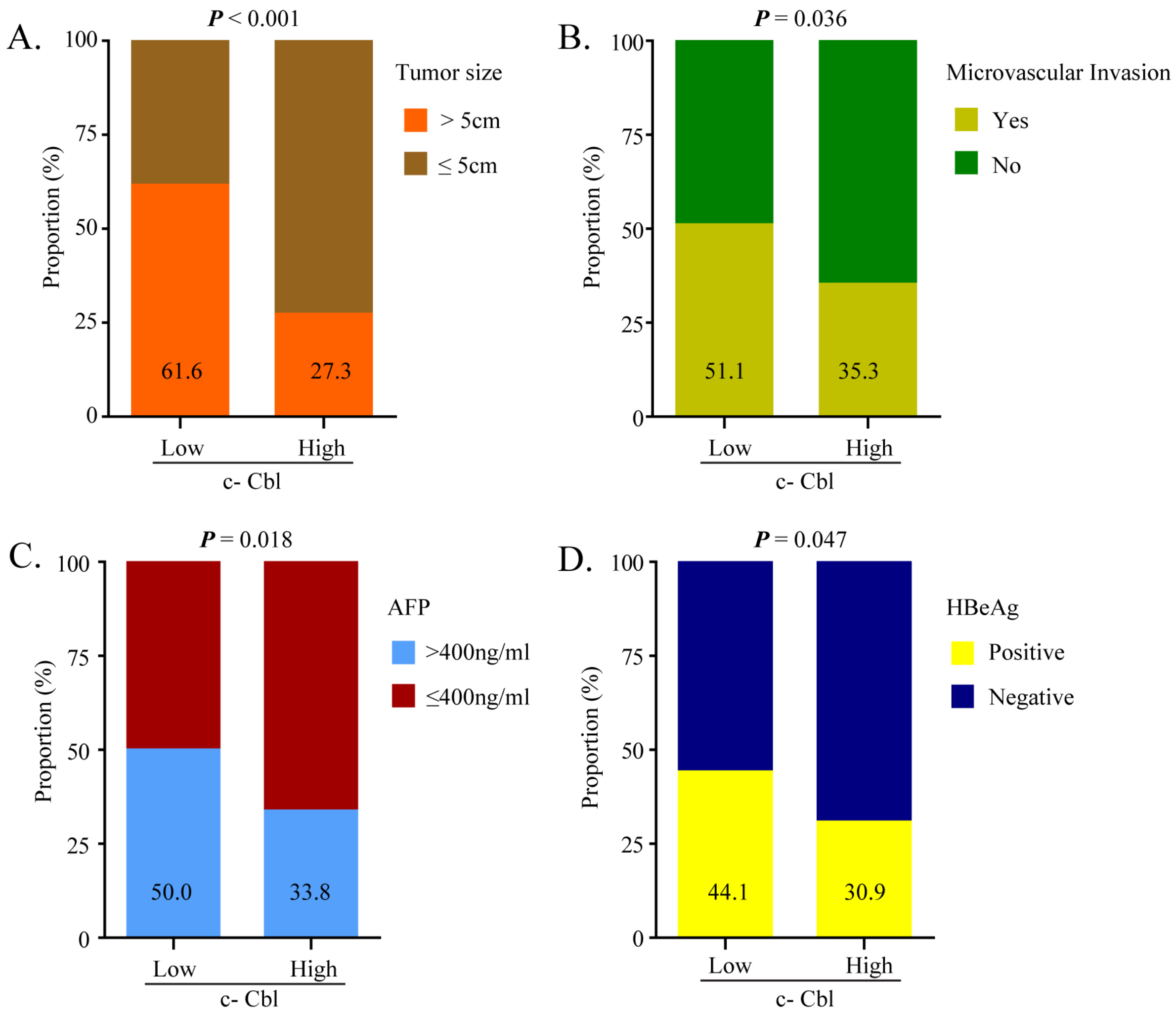

Supplementary Figure S1: The correlation between peritumoral Cbl density and clinical features.

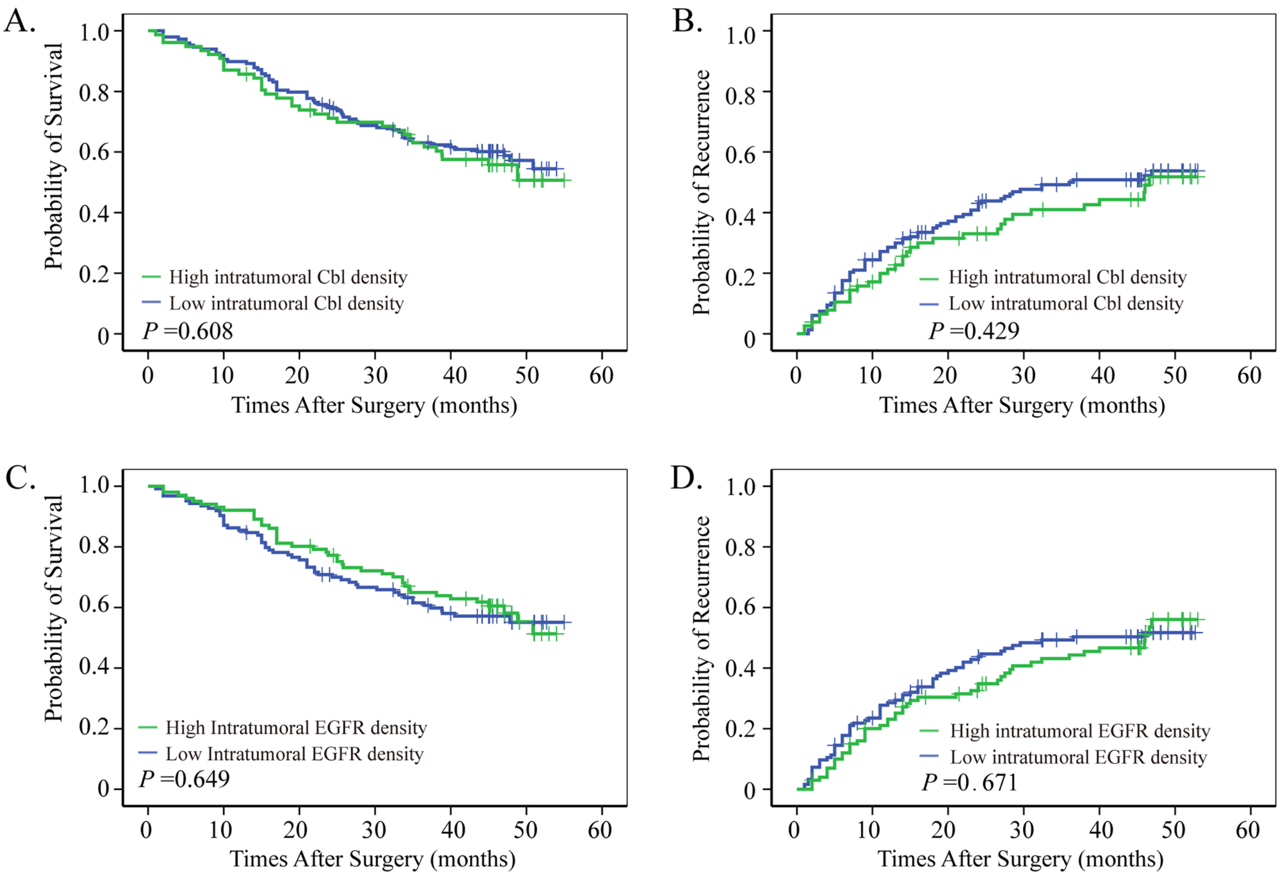

Supplementary Figure S2: The expression of Cbl and EGFR in intratumoral tissue was not significantly associated with clinical outcomes.

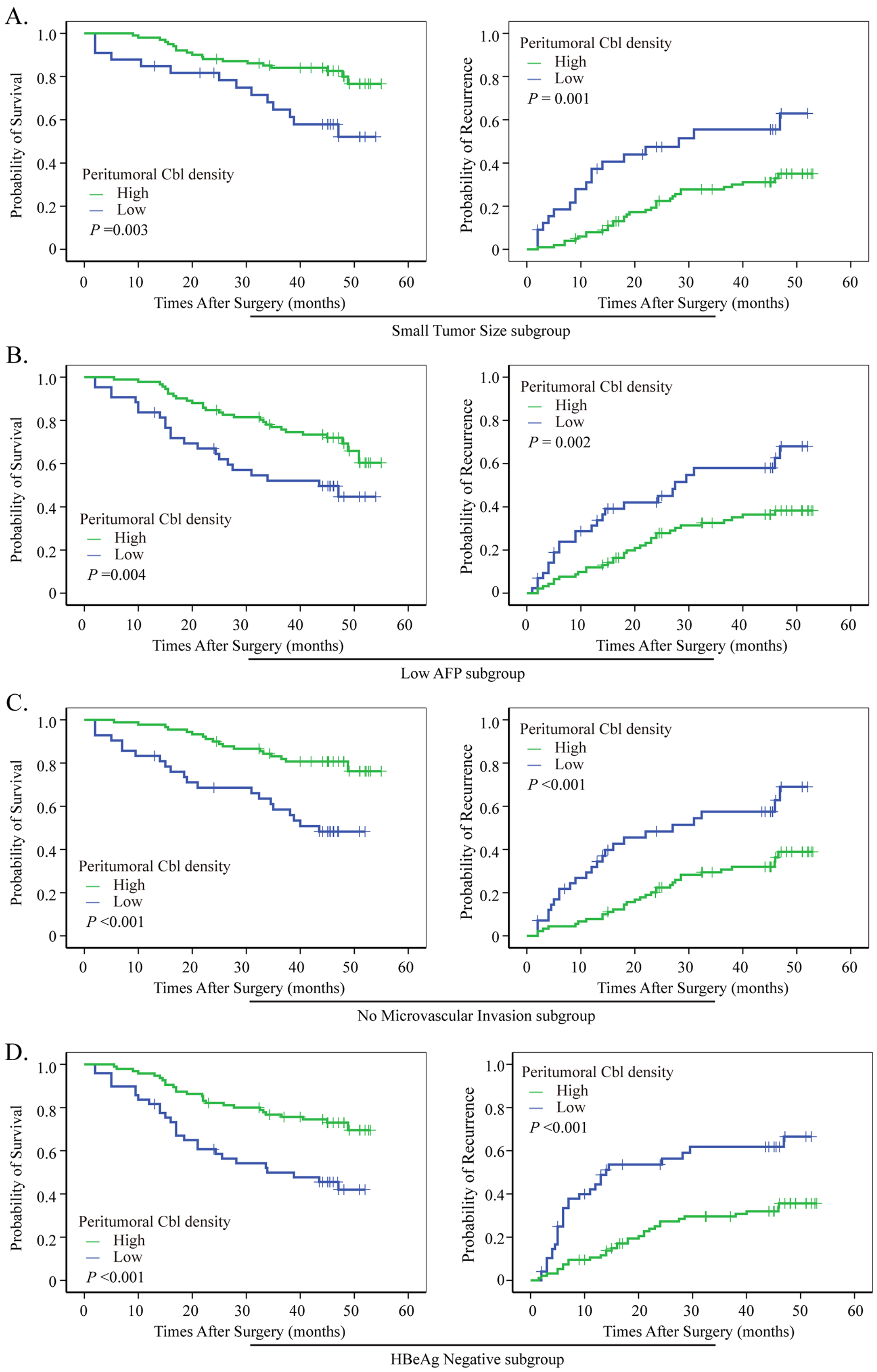

Supplementary Figure S3: The prognostic value of peritumoral Cbl in clinical subgroups.

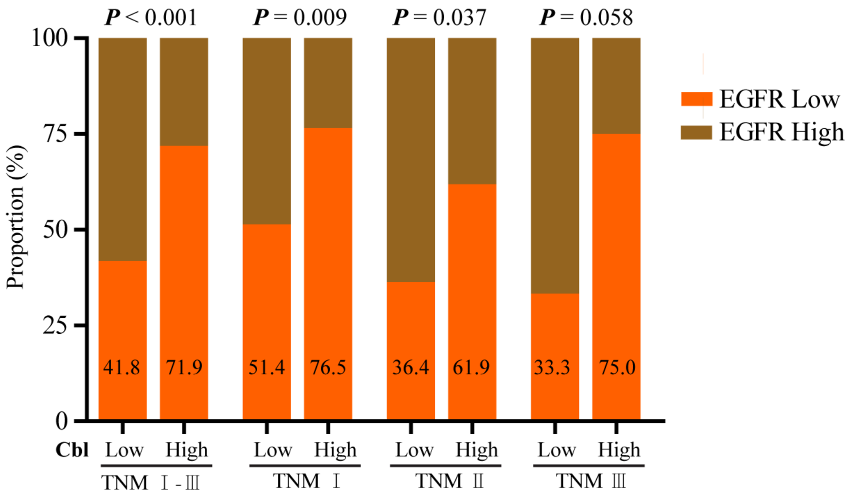

Supplementary Figure S4: Plot representation the correlation between peritumoral Cbl and EGFR.

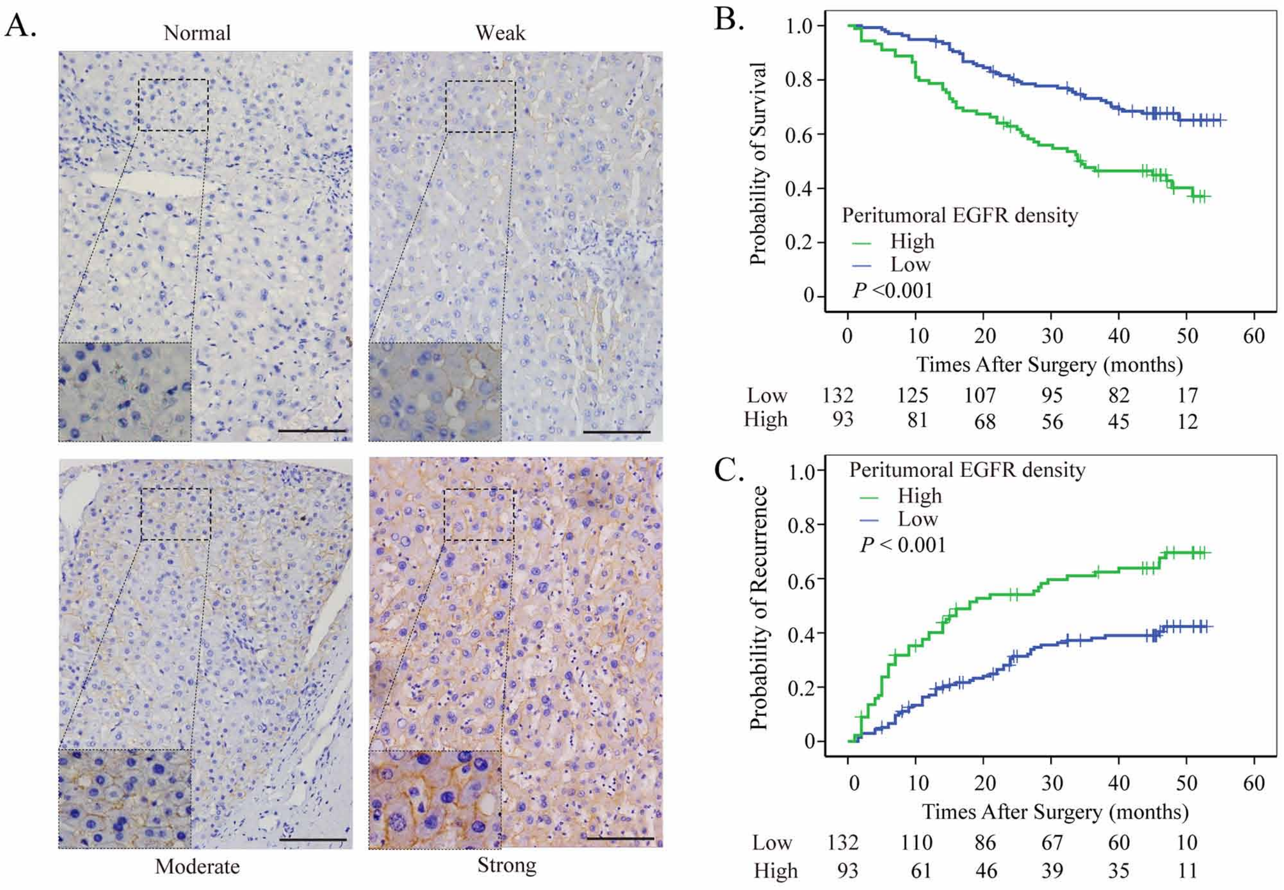

Supplementary Figure S5: Cumulative overall and time-to-recurrence survival curves of patients with low and high peritumoral EGFR expression.

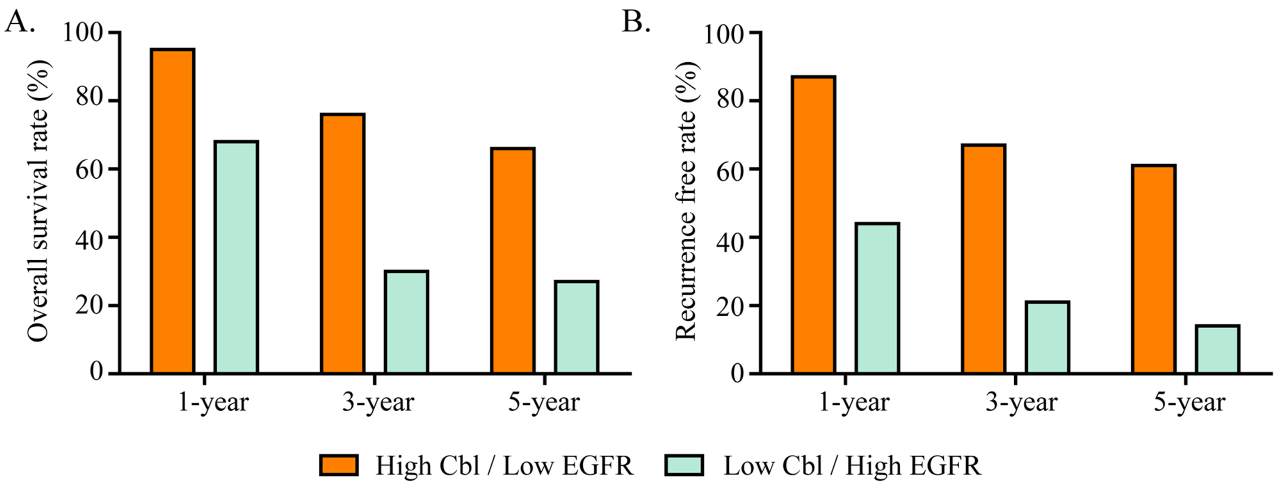

Supplementary Figure S6: The difference of OS and TTR between high Cbl/ low EGFR and low Cbl/ high EGFR.

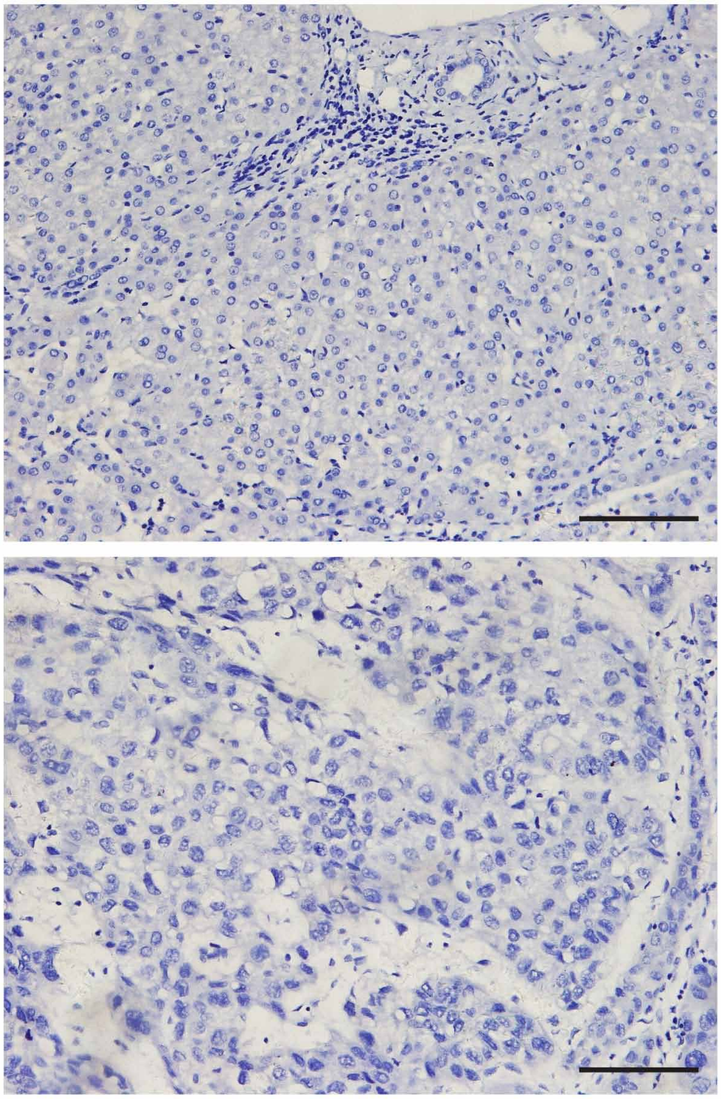

Supplementary Figure S7: Negative controls of immunohistochemistry staining.

Supplementary Table S1: Comparison between full sections and arrayed sections of Cbl in HCC and peritumor tissue

Full section	Arrayed section	Concordance (%)		Value	
Intratumor Cbl	+	81	77	98.2	0.693
	–	144	148		
Peritumor Cbl	+	142	139	98.7	0.770
	–	83	86		

Supplementary Table S2: Relationship between peritumoral and intratumoral Cbl and clinicopathologic features

Variables	Intratumoral Cbl Level*					Peritumoral Cbl Level*				
	High (*n* = 77)		Low (*n* = 148)		*P* value	High (*n* = 139)		Low (*n* = 86)		*P* value
	No. of patients	%	No. of patients	%		No. of patients	%	No. of patients	%	
**Age**, yrs[a]	53.9		51.4		0.115	52		53		0.560
**Sex**					0.141					0.838
Male	71	92.2	125	84.5		122	87.8	74	86.0	
Female	6	7.8	23	15.5		17	12.2	12	14.0	
**Hepatitis B history**					0.148					0.226
Yes	63	81.8	132	89.2		117	84.2	78	90.7	
No	14	18.2	16	10.8		22	15.8	8	9.3	
**HBeAg**					0.770					**0.047**
Positive	29	37.7	52	35.1		43	30.9	38	44.2	
Negative	48	62.3	96	64.9		96	69.1	48	55.8	
**AFP**,U/L[a]	6490		5111		0.491	2804		10074		**0.001**[b]
**ALT**, U/L[a]	46		56		0.277	54		52		0.842
**Liver cirrhosis**					0.066					0.724
Yes	58	75.3	127	85.8		118	84.9	67	77.9	
No	19	24.7	21	14.2		21	15.1	19	22.1	
**Microvascular invasion**					0.064					**0.036**
Yes	25	32.5	68	45.9		49	35.3	44	51.2	
No	52	67.5	80	54.1		90	64.7	42	48.8	
**Tumor size**[a], cm	5.6		5.5		0.904		4.4	7.4		**<0.001**[b]
**Intrahepatic metastasis**					0.842					0.113
Yes	11	14.3	20	13.5		15	10.8	16	18.6	
No	66	85.7	128	86.5		124	89.2	70	81.4	
**Tumor encapsulation**					0.208					0.891
Complete	43	55.8	69	46.7		70	50.4	42	48.8	
No	34	44.2	79	53.4		69	49.6	44	51.2	

(*Continued*)

Variables	Intratumoral Cbl Level*					Peritumoral Cbl Level*				
	High (n = 77)		Low (n = 148)		P value	High (n = 139)		Low (n = 86)		P value
	No. of patients	%	No. of patients	%		No. of patients	%	No. of patients	%	
**Tumor differentiation**					0.375					0.119
I–II	65	84.4	117	79.1		117	84.2	65	75.6	
III–IV	12	15.6	31	20.9		22	15.8	21	24.4	
**TNM stage**					0.380					**0.004**
I	46	59.7	74	50.0		85	61.2	35	40.7	
II	22	28.6	53	35.8		42	30.2	33	38.4	
III	9	11.7	21	12.2		12	8.6	18	20.9	

[a]Independent-samples *t*-test.
[b]Equal variances not assumed.
Abbreviations: AFP: alpha fetoprotein; HBeAg: hepatitis B e antigen.
*Cores of two patients for peritumoral and intratumoral cbl immunostaining were unexpectedly detached from TMA without sufficient tissue to score.

Supplementary Table S3: Clinicalpathologic features of patients from two cohorts

Features	Values/Counts	
	Cohort 1 (n = 227)	Validation cohort(n = 125)
Ages (in years), median (range)	52 (22–80)	51.4 (18–75)
Gender, male/female	198/29	115/10
Preoperative ALT,U/L, median (range)	42.0 (10–806)	46.7 (2–178)
Alpha fetoprotein, median (range)	169 (0–60500)	258 (0–60500)
Liver cirrhosis, yes/no	185/42	97/28
Hepatitis B history, yes/no	196/31	105/20
Hepatitis B e antigen, positive/negative	82/145	30/95
Tumour size, cm, mean ± SD	5.6 ± 3.9	6.8 ± 4.6
Encapsulation, complete/none	112/115	57/68
Tumour differentiation (high/low)	184/43	107/18
Intrahepatic metastasis, yes/no	31/196	25/100
Microvascular invasion, yes/no	94/133	53/72
TNM stage, I/II/III	121/76/30	78/28/19

Supplementary Table S4: Relationship between peritumoral and intratumoral EGFR and clinicalpathologic features

Variables	Intratumoral EGFR Level*					Peritumoral EGFR Level*				
	High (*n* = 101)		Low (*n* = 124)		*P* value	High (*n* = 89)		Low (*n* = 136)		*P* value
	No. of patients	%	No. of patients	%		No. of patients	%	No. of patients	%	
**Age**, yrs[a]	53.5		51.3		0.142	53.2		51.7		0.331
**Sex**					1.000					0.416
Male	88	87.1	108	87.1		80	89.9	116	85.3	
Female	13	12.9	16	12.9		9	10.1	20	14.7	
**Hepatitis B history**					0.560					1.000
Yes	86	85.1	109	87.9		77	86.5	118	86.8	
No	15	14.9	15	12.1		12	13.5	18	13.2	
**HBeAg**					1.000					0.887
Positive	36	35.6	45	36.3		33	37.1	48	35.3	
Negative	65	64.4	79	63.7		56	62.9	88	64.7	
**AFP**,U/L[a]	5450		5691		0.900	3549		8691		**0.016**[b]
**ALT**, U/L[a]	58		49		0.101[b]	58		45		0.125
**Liver cirrhosis**					0.861					0.287
Yes	84	83.2	101	81.5		70	78.7	115	84.6	
No	17	16.8	23	18.5		19	21.3	21	15.4	
**Microvascular invasion**					0.077					**0.006**
Yes	35	34.7	58	46.8		47	52.8	46	33.8	
No	66	65.3	66	53.2		42	47.2	90	66.2	
**Tumor size**[a], cm	5.8		5.2		0.236	4.8		6.7		**0.001**[b]
**Intrahepatic metastasis**					1.000					0.559
Yes	14	13.9	17	13.7		15	16.9	16	11.8	
No	87	86.1	107	86.3		74	83.1	120	88.2	
**Tumor encapsulation**					0.894					1.000
Complete	51	50.5	61	49.2		44	49.4	68	50.0	
No	50	49.5	63	50.8		45	50.6	68	50.0	
**Tumor differentiation**					1.000					0.863
I–II	82	81.2	100	80.6		73	82.0	109	80.1	
III–IV	19	18.8	24	19.4		16	18.0	27	19.9	
**TNM stage**					0.155					**0.017**
I	61	60.4	59	47.6		37	41.6	83	61.1	
II	28	27.7	47	37.9		37	41.6	38	27.9	
III	12	11.9	18	14.5		15	16.8	15	11.0	

[a]Independent-samples *t*-test.
[b]Equal variances not assumed.
Abbreviations: AFP: alpha fetoprotein; HBeAg: hepatitis B e antigen.
*Cores of two patients for peritumoral and intratumoral cbl immunostaining were unexpectedly detached from TMA without sufficient tissue to score.

Supplementary Table S5: Prognostic values of variables for death, recurrence and early recurrence

Variables	Area under curve	95%CI	*P* value
**Death**			
Tumour size	0.715	0.645–0.785	<0.001
Combine Cbl and EGFR	0.678	0.606–0.750	<0.001
Microvascular invasion	0.652	0.579–0.726	<0.001
Peritumoural Cbl	0.643	0.569–0.717	<0.001
Peritumoural EGFR	0.622	0.548–0.697	0.002
Hepatitis B e antigen	0.571	0.495–0.647	0.069
**Recurrence**			
Combine Cbl and EGFR	0.658	0.586–0.729	<0.001
Tumour size	0.627	0.554–0.701	0.001
Peritumoural Cbl	0.622	0.549–0.696	0.002
Peritumoural EGFR	0.618	0.545–0.692	0.002
Hepatitis B e antigen	0.581	0.506–0.656	0.035
Microvascular invasion	0.574	0.500–0.649	0.054
Intrahepatic metastasis	0.537	0.461–0.612	0.342
**Early recurrence**			
Combine Cbl and EGFR	0.660	0.585–0.734	<0.001
Tumour size	0.639	0.564–0.714	<0.001
Peritumoural Cbl	0.629	0.553–0.705	0.001
Microvascular invasion	0.613	0.537–0.689	0.004
Peritumoural EGFR	0.609	0.532–0.685	0.036
Hepatitis B e antigen	0.544	0.466–0.622	0.268
Intrahepatic metastasis	0.538	0.459–0.616	0.342
